# Associations Between Anthropometric Characteristics, Jump Performance and Recorded Tendon-Related Disorders in Youth Volleyball Players

**DOI:** 10.3390/jfmk11030323

**Published:** 2026-08-20

**Authors:** Gaetano Raiola, Giovanni Esposito, Francesco Laterza, Giuseppe Caminiti, Rosario Ceruso, Vincenzo Manzi

**Affiliations:** 1 Research Center of Physical Education and Exercise, Pegaso University, 80143 Naples, Italy; gaetano.raiola@unipegaso.it (G.R.); rosario.ceruso@univr.it (R.C.); 2 Department of Education and Sports Sciences, Pegaso University, 80143 Naples, Italy; francesco.laterza@univr.it (F.L.); vincenzo.manzi@unipegaso.it (V.M.); 3 Department of Neurosciences, Biomedicine and Movement Sciences, University of Verona, 37129 Verona, Italy; 4 Department of Human Science and Promotion of Quality of Life, San Raffaele Open University, 00166 Rome, Italy

**Keywords:** youth volleyball, vertical jump, neuromuscular performance, functional assessment

## Abstract

**Background**: Patellar tendon complaints and other tendon-related disorders are common in jumping sports; however, the relationship between recorded tendon problems, anthropometric characteristics, and neuromuscular performance in youth athletes remains poorly understood. This study explored the association between height, body mass, competitive age category, a retrospectively recorded history of tendon-related disorders, and jumping performance in youth volleyball players. **Methods**: Thirty-three male youth volleyball players competing in the Under-15, Under-17, and Under-19 categories participated in this cross-sectional study. For each athlete, Reach (maximum standing reach height), Vertec (maximum height attained during a maximal vertical jump), and vertical jump height, calculated as the difference between Vertec and Reach, were recorded. The presence or absence of tendon-related disorders was determined retrospectively from medical history information and club documentation. Data were analyzed using a multivariate general linear model, followed by univariate analyses and Bonferroni-adjusted post hoc comparisons. **Results**: Height showed a highly significant effect on the set of performance variables (Wilks’ λ = 0.066; *p* < 0.001), and competitive age category was also significantly associated with performance (Wilks’ λ = 0.655; *p* = 0.049). In contrast, neither body mass (*p* = 0.854) nor the tendon-related disorder variable (*p* = 0.293) demonstrated significant associations. Univariate analyses revealed significant effects of competitive age category on Vertec performance (*p* = 0.018) and vertical jump height (*p* = 0.008), but not on Reach (*p* = 0.542). Bonferroni-adjusted post hoc comparisons showed significant differences between the Under-19 and Under-15 categories for Vertec performance (*p* = 0.017), and between Under-19 and Under-15 (*p* = 0.007) as well as Under-17 and Under-15 (*p* = 0.034) for vertical jump height. **Conclusions**: Jumping performance in youth volleyball appears to be primarily influenced by anthropometric characteristics and developmental factors associated with competitive age category, whereas the dichotomous retrospective classification of tendon-related disorders adopted in the present study did not discriminate performance level. These findings highlight the importance of integrating functional, clinical, and training-load monitoring to better understand athletic development and tendon vulnerability.

## 1. Introduction

Patellar tendon complaints and tendon-related disorders represent common overload-related conditions in sports characterized by a high exposure to ballistic movements and repeated stretch-shortening cycles, such as volleyball, where the tendon is subjected to intense and repetitive mechanical loading [1,2]. Clinically diagnosed patellar tendinopathy is particularly relevant among athletes involved in jumping disciplines, in which tendon pain may impair function, training availability, and the continuity of sports performance [3]. Epidemiological data report a particularly high prevalence of patellar tendon problems among volleyball players, especially in highly competitive settings, confirming the role of sport-specific movements and cumulative load exposure as key determinants of tendon injury risk [4]. In recent years, the conceptual framework for understanding tendinopathies has progressively shifted from a purely structural interpretation toward a multifactorial model in which the relationship between applied load and the load-bearing capacity of the muscle-tendon unit plays a central role [5,6]. Within this perspective, the tendon is no longer viewed as a structure passively exposed to stress, but rather as a dynamic tissue whose response depends on the interaction among mechanical stimuli, biological adaptation, neuromuscular maturation, technical factors, and training organization. Longitudinal evidence in adolescent athletes suggests that elevated tendon strain levels may precede the onset of symptoms, supporting the hypothesis that a mismatch between muscular strength development and tendon adaptation represents a relevant pathogenic mechanism during growth and maturation [7,8]. In the context of youth volleyball, this issue is particularly important, as the progressive increase in performance demands is accompanied by biological maturation and greater exposure to jumping, landing, and rapid changes in direction [9]. The literature on injuries in young volleyball players highlights the growing burden of overuse conditions, suggesting the need to investigate not only injury incidence but also its relationship with anthropometric, functional, and sport-specific performance variables [10]. Within this framework, an integrated analysis of characteristics such as height, body mass, jumping ability, and competitive age category may provide useful insights into the distribution of recorded tendon problems and help identify patterns potentially associated with increased tendon vulnerability [11,12].

From a performance perspective, volleyball provides an especially relevant context for examining the interaction between anthropometry, neuromuscular expression, and tendon-related symptoms because match and training demands include a large number of repeated submaximal and maximal jumps performed under time pressure and in highly variable tactical situations [13,14,15]. In youth categories, these demands are superimposed on ongoing growth-related changes in body dimensions, intermuscular coordination, and muscle-tendon mechanical behavior.

Accordingly, the assessment of vertical jump performance represents a relevant component of athlete evaluation in youth volleyball, provided that the measurement method is feasible within routine training settings. The Vertec vertical jump measurement system is a commonly used field-based method for assessing jump height and has been employed in athletic populations, including volleyball players [16,17]. Its simple setup, limited equipment requirements, short testing procedure, and minimal space requirements facilitate its integration into routine training and athlete-monitoring procedures, particularly when repeated assessments are required. Moreover, vertical jumping represents a fundamental movement demand in volleyball, and jump-specific assessment has been applied to quantify performance and training demands in volleyball settings [18]. Superior jump performance may be associated with greater cumulative tendon loading exposure; however, the present dataset does not allow prospective evaluation of injury onset or confirmation of a clinical diagnosis. Rather, it allows an exploratory comparison of jump performance between athletes with and without a retrospectively recorded history of tendon-related disorders. In addition, the presence of tendon symptoms does not necessarily correspond to a proportional reduction in performance, particularly in young athletes who may continue training and competing despite pain or who present early-stage tendon reactivity without a clear functional deficit [19,20,21]. This complexity highlights the need to interpret jumping performance and retrospectively recorded tendon-related disorders within the same analytical framework while considering developmental and contextual factors that may influence both adaptation and vulnerability [22,23].

Despite the high prevalence of tendon-related disorders in youth volleyball, few studies have simultaneously examined the relationships among retrospectively recorded tendon-related disorders, anthropometric characteristics, competitive age category, and jumping performance. Therefore, the aim of the present study was to investigate whether young male volleyball players with a retrospectively recorded history of tendon-related disorders differed in anthropometric characteristics and jump performance from those without such records while accounting for competitive age category. The tendon-related variable was considered a pragmatic dichotomous indicator derived from medical history information and club documentation, rather than confirmation of current clinically diagnosed patellar tendinopathy. We hypothesized that competitive age category and anthropometric characteristics would be significantly associated with jump performance, whereas the retrospectively recorded tendon-related disorder variable would show limited discriminatory value. Although biological maturation was not directly assessed, it should be considered a possible interpretive factor when discussing differences observed across adolescent competitive age categories.

## 2. Material and Methods

### 2.1. Study Design

The study was conducted using a cross-sectional observational design with descriptive and associative purposes. The primary objective was to analyze the contribution of anthropometric characteristics, competitive age category, and a retrospectively recorded history of tendon-related disorders to vertical jump performance in young volleyball players. All data used in the analyses were derived from the club’s routine performance monitoring activities conducted during the competitive season. No experimental manipulation of variables was introduced, nor were any training protocols specifically implemented for research purposes. Consequently, the present study should be considered non-interventional in nature, and the findings allow only the identification of statistical associations among the variables examined, without supporting causal inferences. The use of data collected within a real-world sporting environment allowed for the maintenance of high levels of ecological validity, enabling the observation of the variables under conditions that were representative of regular competitive practice. Observational research conducted in natural sporting contexts is particularly valuable for examining performance-related phenomena as they occur in everyday training and competition settings. During the competitive season, the athletes followed a structured training program organized according to the specific requirements of the different age categories. The teams typically completed three to four training sessions per week, in addition to participating in the official competitions scheduled by the national federation. Training sessions included an initial phase of general and sport-specific warm-up activities, followed by technical and tactical drills and exercises aimed at developing physical and coordinative abilities.

A relevant component of the training program included physical preparation activities aimed at improving lower-limb strength, muscular power, and explosive performance through general strength training, volleyball-specific strength exercises, and plyometric activities. However, the exact proportion of training time dedicated to these components was not retrospectively quantified and should therefore be interpreted as a qualitative description of the club’s training methodology rather than as a precise workload measure. Although training volume and intensity were adjusted according to age, experience level, and the objectives of each age category, the club’s methodology was based on common principles of load progression, performance monitoring, and long-term athlete development. This organizational consistency helped reduce variability arising from different methodological approaches, thereby enhancing the comparability of the collected data.

### 2.2. Study Participants

The study involved 33 male volleyball players belonging to the youth academy of a competitive volleyball club and regularly engaged in official federation competitions during the 2025–2026 season. The athletes were equally distributed across three age categories: Under-15 (*n* = 11), Under-17 (*n* = 11), and Under-19 (*n* = 11). Although official volleyball rosters generally include 12 players, the final sample included 11 athletes per age category because only eligible players available during the monitoring period were included. All participants consistently took part in the training sessions and competitive activities organized by the club and were registered in their respective age-group championships. Sample size was determined by athlete availability during the data collection period; therefore, a convenience sampling approach was adopted. Given the exploratory nature of the study, no a priori sample size calculation was performed. To be eligible for inclusion, athletes were required to be officially registered with the club, actively participate in competitive activities, regularly attend the training program prescribed for their age category, and be available for the scheduled monitoring session. Athletes were excluded from testing if they had an acute musculoskeletal injury, were currently unable to train or compete fully, had missed recent training because of injury or illness, had a history of knee surgery, reported other lower-limb injuries that could affect jump performance, or experienced pain during the testing procedures. Athletes who reported pain or discomfort during warm-up or testing were not allowed to complete the jump assessment and were referred to the club medical staff according to routine procedures. The inclusion of participants from the same sporting organization provided a relatively homogeneous environment characterized by shared approaches to training planning, load monitoring, and performance development. For each athlete, data were collected on year of birth, competitive age category, height, body mass, jumping performance parameters, and the possible presence of tendon-related disorders that had occurred or been reported during the season. This information was retrieved retrospectively from the club’s medical and technical documentation and should be interpreted as a pragmatic record of tendon-related issues rather than a standardized clinical diagnosis. The available records did not allow a reliable reconstruction of the affected side (right/left knee) or bilateral involvement. Because the documentation was retrospective, the study could not determine whether athletes with previous tendon-related complaints experienced persistent functional limitations, reduced participation, or eventual discontinuation of volleyball activity. Although patellar tendon disorders during adolescence may influence long-term sports participation, the present dataset did not include information on players who discontinued activity or left the club before data collection.

This study is part of the research project entitled “Psychophysical perception and body awareness in school and sports contexts through observational and non-invasive tools”, approved by the Ethics Committee of Pegaso Telematic University (Prot./E 004726, dated 15 July 2025). Before participation, all athletes received age-appropriate information about the aims and procedures of the study. For participants younger than 18 years, written informed consent was obtained from a parent or legal guardian, and the athletes provided assent to participate. Participants aged 18 years provided their own written informed consent.

### 2.3. Data Collection and Measurement

The evaluations were conducted as part of the routine performance monitoring activities scheduled by the sports club in February of the current year. All measurements were performed by the same operators following standardized procedures to ensure consistency and reliability of data collection. Before testing, athletes completed a standardized warm-up consisting of low-intensity running, dynamic mobility exercises, and progressive submaximal vertical jumps to familiarize themselves with the movement pattern and prepare for maximal efforts. Anthropometric parameters were initially recorded. Subsequently, vertical jump performance was assessed using a Vertec vertical jump measurement system. The jump protocol consisted of a countermovement jump (CMJ) performed from an upright standing position with feet approximately shoulder-width apart. Athletes were instructed to perform a rapid downward movement followed by an explosive upward movement, reaching the maximum possible height. Arm swing was permitted, whereas approach steps were not allowed; therefore, all jumps were performed from a stationary position.

Each athlete performed three maximal CMJ trials, separated by 30 s of passive recovery to minimize fatigue effects. Before each attempt, standardized verbal instructions were provided, encouraging athletes to jump as high as possible while maintaining the correct execution technique. A trial was considered valid when the athlete performed the jump without additional steps before take-off, maintained balance upon landing, and achieved clear and detectable contact with the Vertec device at the highest point of the flight phase. Invalid attempts were repeated. Reach was measured as the maximum height achieved by the athlete in a standing position with the dominant arm fully extended overhead. The Vertec value represented the maximum height reached during the maximal vertical jump. Within-session reliability across the three CMJ trials was assessed using an intraclass correlation coefficient (ICC), with 95% confidence intervals, and showed excellent reliability (ICC = 0.98, 95% CI = 0.97–0.99). For each athlete, the mean value of the three valid jump trials was calculated and used for analysis. Vertical jump height was then calculated as the difference between Vertec and Reach, representing an indirect measure of lower-limb explosive capacity. This approach is commonly used in volleyball research because it allows the contribution of anthropometric characteristics to be distinguished from explosive force production. Although absolute Reach and Vertec values provide useful descriptive information regarding athletes’ anthropometric and performance characteristics, these measures are inherently influenced by body stature. Therefore, vertical jump height (calculated as Vertec minus Reach) was considered the primary indicator of explosive lower-limb performance because it reduces the influence of standing height. Consequently, Reach and absolute Vertec values were interpreted as combined anthropometric-performance outcomes, whereas vertical jump height was considered the most specific measure of jumping ability.

The exposure variable was defined as the presence or absence of a documented tendon-related disorder during the competitive season. The classification was performed at the athlete level rather than separately for each knee; therefore, each player was categorized according to whether a history of tendon-related complaints had been documented (yes/no). The variable was coded dichotomously based on retrospective review of medical records and internal club documentation, including documented episodes of patellar tendon pain, tendon-related complaints, or previous tendon-related episodes recorded by the club staff. Because the study relied on retrospective documentation, the available records did not consistently specify the affected side (right or left knee), bilateral involvement, the individual who established the original diagnosis, the diagnostic criteria applied, symptom duration, recurrence status, current symptom status at the time of testing, or whether a standardized clinical examination had been performed. Information regarding imaging procedures or validated patient-reported outcome measures was also not available. Consequently, the tendon-related variable should be interpreted as a pragmatic indicator of documented tendon-related complaints within the club setting rather than as confirmation of clinically diagnosed patellar tendinopathy according to established diagnostic criteria.

The available dataset also did not include information regarding athletes who may have reduced participation or discontinued volleyball activity because of previous tendon-related symptoms. Although tendon-related disorders during adolescence may potentially influence long-term sports participation, the present study was unable to evaluate whether previous complaints affected athlete retention or competitive continuation. Therefore, a possible selection effect related to tendon-related problems cannot be excluded and should be considered when interpreting the findings. The variable was included to explore whether players with documented tendon-related complaints differed in jump performance from those without such documentation.

### 2.4. Statistical Analysis

Data were analyzed using descriptive and inferential statistical procedures. Quantitative variables were summarized using means and standard deviations, while categorical variables were expressed as absolute frequencies and percentages. Before conducting the multivariate analysis, model assumptions were evaluated. Normality of residuals, homogeneity of variance–covariance matrices, and the presence of influential observations were assessed. No relevant violations affecting the interpretation of the model were identified.

To assess the simultaneous effect of the independent variables on the set of performance indicators, a multivariate general linear model was applied. The dependent variables included in the model were Reach, Vertec, and vertical jump height. Given that Reach and absolute Vertec values are partially determined by anthropometric characteristics, particularly stature, the effects of height on these variables were interpreted as expected anatomical relationships rather than as independent indicators of explosive performance. Conversely, vertical jump height was considered the primary performance-related outcome because it represents the actual displacement achieved during the jump after accounting for standing reach. Height and body mass were entered as continuous covariates, while competitive age category and the presence of recorded tendon-related disorders were treated as fixed factors. An interaction term (category × tendon-related disorders) was also included as an exploratory factor to examine whether the association between recorded tendon-related conditions and performance differed across age categories. Considering the limited sample size and the small number of athletes in some category-by-tendon status subgroups, the model was interpreted as exploratory, and interaction effects were interpreted cautiously. Therefore, non-significant findings were not considered evidence of the absence of an association. Multivariate significance was assessed using Wilks’ Lambda, Pillai’s Trace, Hotelling’s Trace, and Roy’s Largest Root. When significant effects were observed, univariate analyses were subsequently performed for each dependent variable while maintaining the same model structure. For each analysis, degrees of freedom (Df), estimated marginal means (adjusted means), and 95% confidence intervals (CI) were reported to provide a more complete description of the effects. Effect size was quantified using partial eta squared (η^2^p), interpreted according to commonly adopted criteria as small (η^2^p ≥ 0.01), medium (η^2^p ≥ 0.06), and large (η^2^p ≥ 0.14). Group differences were further explored through post hoc pairwise comparisons adjusted using the Bonferroni correction. For all analyses, the level of statistical significance was set at *p* < 0.05. Analyses were performed using JASP Open Source Software (version 0.95.0.0; University of Amsterdam, The Netherlands).

## 3. Results

Descriptive statistics of the anthropometric and performance variables by competitive age category are reported in Table 1.

Overall, 13 athletes (39.4%) were classified as having a recorded history of tendon-related disorders during the sports season, whereas 20 athletes (60.6%) had no documented tendon-related problems. This classification was based on retrospective information and should not be interpreted as confirming current clinically diagnosed patellar tendinopathy in all cases. The distribution of recorded tendon-related disorders differed across categories, with the highest proportion observed in the Under-15 and Under-17 groups (both 45.5%), while the Under-19 group showed a lower proportion (27.3%). However, these descriptive differences should be interpreted cautiously due to the limited sample size within each category. Regarding the anthropometric and performance variables, mean values generally increased across competitive age categories, although the pattern was not strictly linear for all variables. The Under-17 and Under-19 groups showed higher values than the Under-15 group for most anthropometric and performance measures (Reach, Vertec, and vertical jump), with the highest mean vertical jump performance observed in the Under-19 group. These differences may reflect the combined influence of biological maturation and accumulated playing experience.

The simultaneous effect of anthropometric variables, competitive age category, and the presence of recorded tendon-related disorders on performance variables was analyzed using a multivariate general linear model (GLM), considering Reach, Vertec, and vertical jump as dependent variables. The results are reported in Table 2.

The multivariate analysis showed a highly significant effect of height on the set of performance variables (Wilks’ λ = 0.066; *p* < 0.001), with a very large effect size (η^2^p = 0.934). This finding was expected given that Reach and absolute Vertec values are directly influenced by stature. Therefore, the effect of height primarily reflects the anatomical contribution to reach-related measurements rather than an independent effect on explosive performance. A significant overall of age category was also observed (Wilks’ λ = 0.655; *p* = 0.049), indicating differences in performance among U15, U17 and U19. In contrast, neither body mass nor the recorded tendon-related disorder variable showed statistically significant effects on the variables considered. Likewise, no significant interaction emerged between category and the recorded tendon-related disorder variable. Univariate analyses were then conducted to identify which specific performance variables were influenced by the factors included in the model. Results of the univariate analyses for the effect of competitive age category are presented in Table 3.

Height was significantly associated with the set of performance variables considered in the multivariate model. The strongest effect was expected for Reach, given the anatomical relationship between stature and standing reach ability. Height also influenced absolute Vertec values, whereas the interpretation of vertical jump height requires caution because this outcome was calculated as Vertec minus Reach and was intended to reduce the influence of body stature. Competitive age category did not show a significant effect on Reach, but it was significantly associated with both Vertec and vertical jump, suggesting that competitive level contributes to jump-related performance independently of anthropometric characteristics. Neither body mass nor the recorded tendon-related disorder variable showed significant effects on any of the dependent variables considered.

To further investigate the differences between categories in the variables that showed a significant category effect, post hoc multiple comparisons with Bonferroni correction were performed. The results are reported in Table 4.

The post hoc analyses confirmed the absence of significant differences between categories for Reach. For Vertec, a significant difference emerged only between Under-19 and Under-15 athletes, with higher values in the more advanced competitive age category, whereas no significant differences were observed between Under-19 and Under-17 or between Under-17 and Under-15. Regarding vertical jump, both Under-19 and Under-17 athletes achieved significantly higher performances than Under-15 athletes, whereas no significant differences emerged between Under-19 and Under-17. Overall, these results indicate that the differences associated with competitive age category mainly concern the explosive ability expressed in jumping, whereas Reach appears to be more closely associated with individual anthropometric characteristics, particularly height.

## 4. Discussion

The findings indicate that, in the examined sample, competitive age category and height were the factors most closely associated with jumping performance in young volleyball players, while the retrospectively recorded tendon-related disorder variable showed no statistically significant relationship with the performance measures analyzed. The effect of competitive age category was particularly evident for Vertec and vertical jump height, the variables most directly related to explosive performance, with jumping performance tending to improve across competitive age categories. This trend is consistent with previous studies reporting progressive improvements in jumping ability across adolescent volleyball players as a consequence of maturation and accumulated training experience [24,25]. Chronological age is typically accompanied by biological maturation, increased muscular strength, better intersegmental coordination, and greater exposure to sport-specific training [26,27]. Therefore, differences between competitive age categories may reflect the combined influence of developmental and training-related processes occurring throughout adolescence. Together, these factors may enhance the ability to generate force rapidly and apply it effectively during jumping tasks.

The pronounced effect of height on the set of dependent variables, particularly on Reach, appears physiologically plausible, as stature directly influences reaching ability and contributes substantially to the absolute height attained during a jump [28]. Similar observations have been reported in volleyball research, where anthropometric characteristics, particularly stature and limb length, substantially influence absolute reach measurements independently of lower-limb explosive capacity [29,30]. The finding that Reach did not differ significantly between categories after accounting for covariates suggests that this measure is more closely associated with individual anthropometric characteristics rather than the level of explosive development. In contrast, Vertec and especially vertical jump height appear to capture more sensitively the performance differences associated with competitive age category. This distinction highlights the importance of considering anthropometric characteristics when interpreting absolute jump-related measures in adolescent athletes.

The absence of a significant effect of body mass may indicate that absolute body mass alone is insufficient to explain performance differences in this population. Body mass represents a general anthropometric characteristic and does not provide information regarding the qualitative components of body composition or functional characteristics that may contribute to jumping ability. Accordingly, the large contribution of height should not be interpreted as evidence that taller athletes necessarily possess superior explosive capacity, but rather as a reflection of the mathematical and anatomical relationship between stature and absolute reaching performance.

A particularly relevant finding concerns the absence of significant differences between athletes classified with and without recorded tendon-related disorders in the performance measures analyzed. However, this result should be interpreted in light of the simplified retrospective classification adopted in the present study, which was based on medical history information and club documentation and did not include standardized clinical examination, symptom severity, functional limitation, pain intensity, imaging findings, or confirmation of current patellar tendinopathy. Therefore, the absence of a statistical association should be interpreted as evidence that the classification used in the present investigation did not discriminate jumping performance, rather than evidence that tendon-related conditions are unrelated to performance capacity.

This finding is consistent with the previous literature suggesting that the relationship between tendon-related conditions and performance may be complex and not necessarily reflected by immediate reductions in physical performance, particularly when athletes continue participating in sport activities [31,32]. However, the present results should be interpreted within the context of the retrospective classification adopted, which allowed identification of recorded tendon-related history but not differentiation of clinical severity or current functional status. Moreover, the relatively small sample size and the absence of information regarding symptom duration, affected side, biological maturation, and training-load exposure further limit the interpretation of the observed lack of association between tendon-related disorder status and jumping performance. At the same time, the absence of a statistical association does not imply that tendon-related disorders lack clinical relevance, as their impact may extend beyond the performance variables considered in this investigation.

These findings support the view that jumping performance in youth volleyball should be interpreted within a multifactorial framework, in which biological development, anthropometric characteristics, and sport-specific demands interact dynamically [33]. Adolescence is a critical phase for physical development, during which changes in body dimensions, strength, coordination, and athletic experience may influence performance outcomes [34]. Accordingly, the higher jump values observed in the more advanced categories likely reflect differences in competitive level and developmental stage rather than isolated indicators of superior athletic capacity. From an applied perspective, periodic jump-performance monitoring may help track performance development across categories, but outcomes should be interpreted alongside information on maturation and training context. From a practical standpoint, these results reinforce the importance of interpreting jump-test outcomes in context rather than as isolated indicators of readiness or risk [35,36]. For coaches and practitioners working in youth volleyball, higher jump values in older categories can be considered a positive marker of performance development across age categories. However, these findings should be interpreted considering the multifactorial nature of athletic development and tendon-related conditions. Previous research has highlighted that repeated sport-specific activities, including jumping actions and high-intensity movements, may contribute to the mechanical demands experienced by young volleyball players [37,38]. Therefore, the interpretation of jump-performance outcomes may benefit from an integrated perspective that considers both performance development and athlete health. Accordingly, future monitoring approaches may benefit from combining jump-performance assessment with additional information related to training exposure, symptoms, recovery status, and maturation-related factors, as suggested by previous literature [39]. This integrated approach is especially relevant in adolescent athletes, in whom rapid performance improvements may occur alongside complex developmental changes. From a preventive standpoint, current evidence supports balancing performance development with progressive management of sport demands through appropriate training organization, recovery, and early identification of potential concerns [40,41,42]. This perspective is consistent with recent work in youth team-sport athletes combining anthropometric assessment with conditional and explosive performance monitoring to interpret developmental differences across age categories and training contexts [43,44].

The results should be interpreted in light of several important limitations. First, the sample size was relatively small (*n* = 33), participants were recruited from a single sporting context, and convenience sampling was used, limiting the generalizability of the findings. An additional limitation is the absence of an a priori sample size calculation. Although the study was exploratory and conducted in a real-world setting, the limited sample may have reduced the ability to detect small-to-moderate associations, particularly for tendon-related variables. Consequently, the findings should be considered preliminary and hypothesis-generating. This limitation is further emphasized by the inclusion of multiple factors and covariates despite the limited number of observations, increasing the risk of unstable estimates and Type II errors. Second, the cross-sectional design precludes causal inference and does not allow determination of the temporal direction of the associations observed between anthropometric characteristics, jumping performance, and tendon-related disorders. Another limitation is the absence of direct measures of biological maturation. Within the age groups considered, competitive age category may reflect not only chronological age and training experience but also substantial interindividual differences in maturational status, influencing height, strength, coordination, and jumping ability. Similarly, body mass alone does not distinguish between body composition components, and more comprehensive anthropometric assessments may provide additional insight. An important limitation concerns the tendon-related disorder variable, which was classified dichotomously from medical history and internal club records. Although this reflects real-world practice, the available documentation did not consistently specify the diagnosing clinician, diagnostic criteria, symptom duration, recurrence, or the use of standardized clinical assessment procedures. It also did not allow identification of the affected side, pain intensity, clinical severity, disease stage, functional impact, or asymptomatic tendon abnormalities [45,46,47]. Furthermore, the retrospective classification could not distinguish between athletes with resolved tendon-related complaints and those with ongoing symptoms at the time of testing, conditions that may differently influence jumping performance. Therefore, the adopted classification should be interpreted as an indicator of recorded tendon-related history rather than confirmation of clinically diagnosed patellar tendinopathy, and it does not differentiate previous episodes, current symptoms, or clinical severity.

The present study focused on anthropometric characteristics, jump performance, competitive age category, and recorded tendon-related status. Additional clinical and training-related variables may contribute to a more comprehensive understanding of the factors influencing performance development and tendon-related conditions in youth volleyball players. Finally, although the jump tests used are ecologically valid and widely applied, they provide only indirect measures of explosive performance and do not capture the mechanical or coordinative strategies underlying jumping ability. Future multicenter longitudinal studies with larger samples should include direct measures of biological maturation, body composition, detailed clinical characterization of tendon-related conditions, complementary performance assessments, and systematic monitoring of training-related variables to better understand how developmental factors, anthropometric characteristics, performance progression, and tendon-related conditions interact throughout youth volleyball participation.

## 5. Conclusions

The results indicate that competitive age category and height were the variables most strongly associated with jump performance, whereas body mass and the retrospectively recorded tendon-related disorder variable showed no statistically significant associations with the measures considered. Age category-related differences were mainly observed for Vertec and vertical jump performance, with higher values in the more advanced categories, while Reach appeared more closely linked to individual anthropometric characteristics. Overall, the findings suggest that jumping ability in youth volleyball is associated with competitive age category and anthropometric features, consistent with evidence describing performance development during adolescence. For coaches, these findings support the use of periodic jump-performance monitoring as a simple complementary tool for tracking individual development, particularly when players transition between competitive categories. Importantly, jump values should be interpreted relative to the athlete’s age, competitive age category, and anthropometric characteristics rather than used as isolated criteria for comparing players or evaluating developmental progress. Coaches may also use repeated measurements to identify individual performance trends over time and to inform the progressive adjustment of physical and volleyball-specific training demands. However, the dichotomous retrospective classification of tendon-related disorders adopted in this study did not differentiate athletes according to jump-performance outcomes Therefore, the absence of an observed association should not be interpreted as an indication that jump testing can replace clinical monitoring. Coaches should consider athletes’ symptoms, functional status, training exposure, and developmental stage when monitoring players with a history of tendon-related complaints and should refer athletes for appropriate clinical assessment when persistent symptoms are present. Given the exploratory, cross-sectional design and the inclusion of only 33 male youth volleyball players from a single club, these findings should be considered preliminary and should not be generalized without confirmation from larger multicenter longitudinal studies including more comprehensive biological, clinical, and training-load variables.

## Figures and Tables

**Table 1:** Descriptive statistics by competitive age category.

Variable	U19 (*n* = 11)	U17 (*n* = 11)	U15 (*n* = 11)
Age (years)	18.1 ± 0.6	16.8 ± 0.4	14.4 ± 0.3
Body mass (kg)	81.00 ± 9.03	81.27 ± 3.69	69.09 ± 8.50
Height (cm)	189.00 ± 14.20	190.73 ± 5.75	175.64 ± 9.67
Reach (cm)	246.67 ± 19.76	249.36 ± 9.41	230.09 ± 13.39
Vertec (cm)	329.89 ± 17.31	328.73 ± 6.53	305.00 ± 11.32
Vertical jump (cm)	83.22 ± 6.83	79.36 ± 9.88	74.91 ± 5.30
Recorded tendon-related disorders, *n* (%)	3 (27.3%)	5 (45.5%)	5 (45.5%)
Absence of recorded tendon-related disorders, *n* (%)	8 (72.7%)	6 (54.5%)	6 (54.5%)

**Table 2:** Results of the multivariate analysis.

Effect	Wilks’ λ	F	Df	*p*	η^2^p	95% CI
Body mass	0.986	0.159	2, 24	0.854	0.014	[0.000, 0.149]
Height	0.066	156.457	2, 24	<0.001	0.934	[0.869, 0.961]
Category	0.655	2.591	4, 48	0.049	0.191	[0.000, 0.354]
Recorded tendon-related disorders	0.894	1.300	2, 24	0.293	0.106	[0.000, 0.344]

**Table 3:** Univariate analyses for Reach, Vertec, and vertical jump.

Variable	Df	F	*p*	η^2^p	U19 Adj. Mean (95% CI)	U17 Adj. Mean (95% CI)	U15 Adj. Mean (95% CI)
Reach	2, 25	0.629	0.542	0.052	240.96 (238.82–243.10)	241.33 (239.33–243.32)	242.73 (240.44–245.03)
Vertec	2, 25	4.826	0.018	0.296	325.41 (320.64–330.17)	323.20 (318.75–327.65)	314.48 (309.37–319.60)
Vertical Jump	2, 25	6.053	0.008	0.345	84.44 (79.50–89.39)	81.87 (77.26–86.49)	71.75 (66.45–77.06)

Note. Statistically significant effects are highlighted where applicable. η^2^p = partial eta squared.

**Table 4:** Post hoc multiple comparisons (Bonferroni) between categories.

Variable	U19 vs. U17	U19 vs. U15	U17 vs. U15
Reach	1.000	0.847	1.000
Vertec	1.000	0.017 *	0.066
Vertical Jump	1.000	0.007 *	0.034 *

Note. Values represent Bonferroni-adjusted *p*-values. * *p* < 0.05.

## Data Availability

The original contributions presented in this study are included in the article. Further inquiries can be directed to the corresponding author.
